# Testing a Home Solution for Preparing Young Children for an Awake MRI: A Promising Smartphone Application

**DOI:** 10.3390/children10121866

**Published:** 2023-11-28

**Authors:** Sam Geuens, Jurgen Lemiere, Jessica Nijs, Marlies Treunen, Michael Aertsen, Jaan Toelen, Greet Pauwels, Kate Sauer, Marlies Potoms, Sofie Van Cauter, Leen Wouters, Kathrin Hohlbaum, Marie Sjölinder, Olov Ståhl, Gunnar Buyse, Philippe Demaerel, Barbara Weyn

**Affiliations:** 1 University Hospitals Leuven, 3000 Leuven, Belgiumjessica.nijs@uzleuven.be (J.N.); marlies.treunen@kuleuven.be (M.T.);; 2 AZ Sint-Jan, 8000 Brugge, Belgium; 3 Jessa Ziekenhuis, 3500 Hasselt, Belgium; 4 Department Medical Imaging, Ziekenhuis Oost-Limburg, 3600 Genk, Belgium; 5 Centre for Translational Psychological Research TRACE, Ziekenhuis Oost-Limburg, 3600 Genk, Belgium; 6 Ziekenhuis Oost-Limburg, 3600 Genk, Belgium; 7 RWTH Aachen University, 52062 Aachen, Germany; 8 Research Institutes of Sweden (RISE), 103 33 Stockholm, Sweden; marie.sjolinder@ri.se (M.S.);; 9 ESAT-PSI, KU Leuven, 3000 Leuven, Belgium

**Keywords:** pediatric medical imaging, digital health, MRI

## Abstract

Thanks to its non-invasive nature and high-resolution imaging capabilities, magnetic resonance imaging (MRI) is a valuable diagnostic tool for pediatric patients. However, the fear and anxiety experienced by young children during MRI scans often result in suboptimal image quality and the need for sedation/anesthesia. This study aimed to evaluate the effect of a smartphone application called COSMO@home to prepare children for MRI scans to reduce the need for sedation or general anesthesia. The COSMO@home app was developed incorporating mini-games and an engaging storyline to prepare children for learning goals related to the MRI procedure. A multicenter study was conducted involving four hospitals in Belgium. Eligible children aged 4–10 years were prepared with the COSMO@home app at home. Baseline, pre-scan, and post-scan questionnaires measured anxiety evolution in two age groups (4–6 years and 7–10 years). Eighty-two children participated in the study, with 95% obtaining high-quality MRI images. The app was well-received by children and parents, with minimal technical difficulties reported. In the 4–6-year-old group (N = 33), there was a significant difference between baseline and pre-scan parent-reported anxiety scores, indicating an increase in anxiety levels prior to the scan. In the 7–10-year-old group (N = 49), no significant differences were observed between baseline and pre-scan parent-reported anxiety scores. Overall, the COSMO@home app proved to be useful in preparing children for MRI scans, with high satisfaction rates and successful image outcomes across different hospitals. The app, combined with minimal face-to-face guidance on the day of the scan, showed the potential to replace or assist traditional face-to-face training methods. This innovative approach has the potential to reduce the need for sedation or general anesthesia during pediatric MRI scans and its associated risks and improve patient experience.

## 1. Introduction

Magnetic resonance imaging (MRI) techniques offer high-resolution images of soft body tissue without radiation exposure, which increases their demand in medical practice for both adults and children [1]. However, obtaining high-quality images from pediatric patients can be challenging due to their fear of unfamiliar surroundings, loud noises, and the narrow scanner opening, which results in anxiety-induced movements and, ultimately, low-quality images [1].

To optimize child cooperation, healthcare providers and researchers aim to enhance the child-friendliness of MRI procedures through the development of tailored pediatric MRI protocols, accelerated imaging sequences, advanced noise reduction techniques, mood customization options, as well as the integration of entertainment systems [2,3]. Moreover, nonpharmacological interventions rooted in educational and behavioral strategies have been implemented to effectively prepare children for MRI examinations, leading to favorable outcomes even in young children [2,4,5,6]. These preparatory methods employ various tools and content, all of which share the common objective of familiarizing children with the characteristics of an MRI scanner (including the noise, confined space, duration, and appearance) and training them to remain still for extended periods [7]. However successful these preparation protocols may be, their implementation necessitates specific and sometimes costly equipment, such as booklets, instruction movies, mock scanners, or virtual reality goggles, and are typically conducted by dedicated child specialists, which demands a substantial time investment from highly specialized professionals [2,8,9,10].

Not all healthcare settings are equipped with the necessary facilities to provide specialized pediatric MRI approaches. Consequently, sedation is commonly employed as a standard practice to address the challenge of suboptimal imaging outcomes in pediatric MRI scans and enhance diagnostic effectiveness [11]. The level of sedation can range from light anxiolytics to general anesthesia (GA), with GA being the preferred option for young children and those at a higher risk of motion artifacts [12]. However, it is important to acknowledge that sedation and anesthesia procedures carry inherent health-related risks, including the potential for under- or oversedation, respiratory depression, cardiovascular events, and allergic reactions [11,13]. Additionally, the use of sedation or GA in MRI procedures leads to an increase in safety reports, disruptions to workflow, scheduling conflicts, longer hospital stays for both children and parents, and higher visit costs compared with awake MRI procedures [11,14,15].

Despite the aforementioned drawbacks, the utilization of sedation in MRI procedures is still a common practice, particularly among young children [16]. Reported sedation rates range from 74.6% to 91.1% for children below the age of seven undergoing an MRI scan [16]. Notably, the decrease in sedation rates after the age of seven suggests that older children demonstrate a greater capacity for cooperation during MRI investigations. This phenomenon may be attributed to the more advanced development of cognitive compensatory strategies in older children compared with their younger counterparts [17], rather than a reduction in discomfort or anxiety experienced by older children [18]. These findings underscore the importance of developing innovative MRI preparation strategies that specifically target the training of cognitive control techniques in young children while also addressing anxiety-related concerns across all age groups. By doing so, the aim is to minimize the reliance on sedation in pediatric MRI scanning. As new technologies are proven to be valuable [7,19], this study aims to examine the potential of a new smartphone application as a means to prepare children at home for upcoming MRI scans, with the ultimate goal of reducing the need for sedation or general anesthesia (GA) while minimizing human involvement. To achieve this, an international interdisciplinary consortium comprising software engineers, pediatricians, pediatric radiologists, and researchers was established as part of the EIT Health COSMO@home project (https://eithealth.eu/product-service/cosmohome/, acessed on 1 January 2019), which received funding from EIT Health.

The primary objective of this study was to investigate the evolution of anxiety levels before and after the smartphone preparation at home for MRI scans in two distinct age groups: young children (4–6 years old) and older children (7–10 years old). Additionally, this study aimed to explore the outcomes during and after the MRI scans in both age groups.

## 2. Materials and Methods

### 2.1. App Development and App Design

Over the past decade, the radiology department at University Hospitals Leuven has developed a face-to-face training protocol known as the COSMO protocol to prepare young children for MRI scans [20]. This protocol combines storytelling, exposure exercises, information provision, and relaxation techniques. Children eligible for the training were scheduled for designated “COSMO slots” and received personalized face-to-face preparation from a highly experienced trainer approximately one hour before their MRI scan. However, in an effort to reduce reliance on the trainer and make the process more scalable, an EU consortium investigated the potential of a digital app to partially replace or assist the trainer by enabling home-based preparation through serious gameplay.

In the first step, the learning goals (things children should learn to enter the MRI) were determined by analyzing the framework used in an existing preparation app and by analyzing the COSMO protocol in workshops with experts [21]. Seven learning goals were identified and are listed in Table 1. In the second step, these learning goals were translated into minigames and inserted into a digital game app. The process was then iterated during several test runs with small test groups of 4–5 users. The details of this design journey are described by Sjölinder et al. (2021) [22].

**Figure 1:**
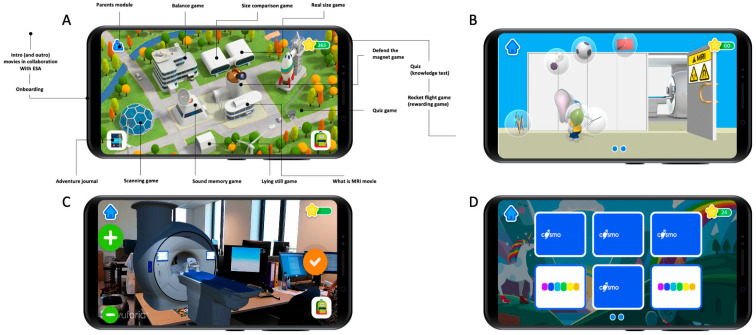
App design and look. (**A**) Overview of the space campus with all the buildings and minigames. (**B**) Screenshot of the “Defend the magnet” minigame, where children have to prevent metal objects from flying into the MRI room by tapping on them. (**C**) “Real size game”. Using augmented reality, children can project the MRI scanner in their room and scale it until they think it is “real-sized”. (**D**) “Sound memory game” minigame, where children need to pair the same MRI sounds by simultaneously turning two identical sound cards.

The app’s homepage features a floor plan of a space campus with several buildings, as depicted in Figure 1. Each building contains a hidden minigame that teaches a specific learning goal (Table 1). The app presents a storyline where players take on the role of aspiring astronauts who can graduate and embark on a rocket journey to another planet by playing the minigames. A digital character, Ollie the Elephant [21], serves as a guide at the training camp, providing instructions. By successfully completing the minigames, players collect rocket parts to build their own spaceship and ultimately engage in a reward game after each round. As the game progresses, the training missions become more challenging. The mini-games are designed to explain all aspects of an MRI procedure and effectively address the defined learning goals. Before accessing the reward game, users are required to complete a mini-quiz to assess their acquired knowledge.

### 2.2. Study Design and Procedure

The final version was used in a multicenter study with four different hospitals located in Flanders, Belgium: the University Hospitals Leuven, the regional Hospital Sint-Jan in Bruges, the regional Hospital Jessa in Hasselt, and the regional Hospital Oost-Limburg in Genk. The hospitals were selected based on the presence of pediatric and radiology departments equipped with MRI scanners offering in-bore patient experience options. Besides the University Hospital Leuven, the other three hospitals had little or no experience in preparing children for an MRI scan. As a result, most children below the age of seven in these hospitals were sedated. The ethics committee of the University Hospital Leuven approved this study and the central protocol (s64701), which was confirmed by the local ethics committees of the other three hospitals. 

Pediatric consultants assessed the eligibility of children based on specific inclusion criteria: (1) age between 4 and 10 years; (2) no significant developmental disorder; (3) no previous awake MRI scan with preparation with the COSMO protocol; (4) native Dutch-speaking; and (5) no other severe medical contraindication.

**Figure 2:**
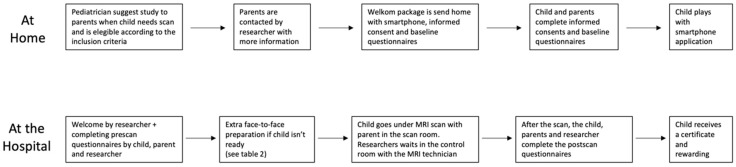
Flowchart of study design.

Eligible children and their parents were introduced to this study (Figure 2), and if consent was obtained, a welcome package containing the smartphone with the COSMO@HOME app, an information folder, props for minigames, and a baseline questionnaire was sent to their homes. Both parents and children provided written consent and completed the baseline questionnaires before using the app. On the day of the scheduled MRI scan, parents brought the signed consent, completed questionnaires, smartphone, and props to the appointment. The children and parents were greeted by an experienced child specialist who conducted a standardized welcome interview. Pre-scan questionnaires, a mini-quiz in the app, and observations by the researcher were used to determine whether a child required additional face-to-face preparation or could proceed directly to the scan. The criteria for proceeding without additional preparation are outlined in Table 2. If additional preparation was needed, it was provided for critical points before the child was permitted to undergo the scan.

During the MRI scan, the child was accompanied by a parent, and a researcher monitored the procedure from the control room. The child chose which movie to watch during the scan. The researcher noted any interruptions, motion artifacts, or deviations from the planned scan duration. If the scan was aborted due to panic or lack of cooperation from the child, it was rescheduled with sedation or general anesthesia in consultation with the pediatrician.

After the scan, the child and parent completed post-scan questionnaires, and the child received a certificate and a small toy as a reward.

### 2.3. Data Acquisition

#### 2.3.1. Questionnaires

In this study, we developed a comprehensive set of questionnaires to measure various aspects related to user preferences, anxiety levels, and willingness to undergo MRI. The development process followed established guidelines for survey development in the scientific research [23]. Our approach included expert focus groups, a literature review, item development, expert validation, and pilot testing.

To ensure the suitability of the questionnaires for different age groups, we employed 6-point visual analog scales (VASs) with pictorial support for children and 10-point Likert scales for parents and researchers [24]. We collected data at multiple time points to capture changes in responses and experiences over time.

The baseline questionnaire gathered information from parents about their child’s scan history and estimated anxiety levels for the upcoming MRI scan. The pre-scan questionnaires for children focused on their preferences for the app, while parents and researchers rated the child’s anxiety levels before the scan. During the scan procedure, researchers gathered additional information such as the preparation time, emotional state, scan duration, scan quality, interruptions, and movements of the child.

In the post-scan questionnaire, children and parents rated their satisfaction with the app as a preparation tool and their willingness to use it again. Parents also rated their child’s anxiety during the scan, while researchers assessed the child’s anxiety, the success of the MRI procedure, and how effectively the app aided the child. 

#### 2.3.2. Log Data

Upon opening the app, all activities were automatically logged. Data regarding the duration and frequency of playing the different games were logged, and the child’s interactions with the games were monitored to determine the child’s evolution while playing. A summary file of these data could be extracted from the smartphone and imported into the database. 

### 2.4. Data Analysis

Data from questionnaires were entered into a database, as well as log data. SPSS Statistics version 28.0.0.0 was used for all analyses, including frequency and descriptive statistics. A Shapiro–Wilk test of normality was conducted to determine whether the anxiety and willingness data were normally distributed. Paired-sample *t*-tests were used to compare means between time points. Independent-sample *t*-tests were used to compare means between groups. A repeated measures ANOVA was used to examine an interaction effect of two age groups between different time points. Results were considered significant at an 0.05 confidence level.

## 3. Results

### 3.1. Study Population and General Findings

**Figure 3:**
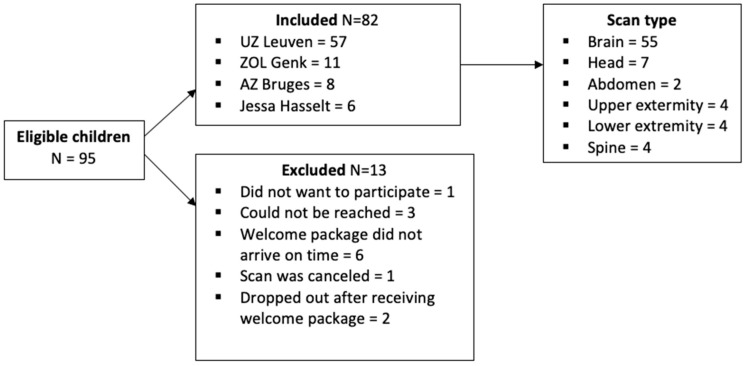
Flowchart of inclusions and exclusions.

A total of 95 children were found eligible by the prescribing pediatrician, but 13 were excluded for various reasons (Figure 3). The main descriptive results of the 82 included children are displayed in Table 3. Of those who had a previous MRI experience, only 12 (average age: 6.1, SD = 12.3, and range = 6–10 years) had an MRI scan without sedation or anesthesia. None of these children had previously received the COSMO protocol as preparation.

Parents reported that their children could play with the app without help (with an average score of 8.3 out of 10; SD = 1.9), and no major technical difficulties occurred. Yet, 21 children (26%) experienced one or two minor technical problems. Only 9% experienced these problems more than three times. In zero cases, these problems prevented the child from continuing to play with the app. 

During the additional training sessions, lying still was the learning goal that needed to be practiced the most, followed by loud noises, the duration of the scan, the MRI size, and metals. 

Most children were at ease when placed in the MRI; however, six children (five from the 4–6 year group, and one from the 7–10 year group) panicked while being positioned on the scanner table. In three children in this group, the scanning procedure could be started after additional comfort, but the scanning of one of these children was aborted in the process since the child became very upset. In three children, the scanning procedure could not be started. These four patients were scheduled for a scan under sedation.

### 3.2. Anxiety and Willingness Evolution

Independent-sample *t*-tests were conducted to examine anxiety scores between the two age groups (4–6 years old and 7–10 years old) based on baseline, pre-scan, and post-scan parent-reported anxiety scores. The results show no significant differences in anxiety scores between the two age groups in baseline (t(80) = 1.77; *p* = 0.081), pre-scan (t(79) = −0.638; *p* = 0.53), or post-scan (t(78) = −1.11; *p* = 0.91) parent-reported anxiety scores.

Furthermore, a paired-sample *t*-test was conducted to compare baseline and pre-scan parent-reported anxiety scores within each age group (Table 4). In the 4–6-year-old group, there was a significant difference between baseline and pre-scan parent-reported anxiety scores (t(31) = 3.52; *p* < 0.001), indicating an increase in anxiety levels prior to the scan. However, in the 7–10-year-old group, no significant difference was observed between baseline and pre-scan parent-reported anxiety scores (t(48) = 1.27; *p* = 0.17), suggesting a relatively stable level of anxiety in this age group before the scan.

Additionally, both age groups showed a significant reduction in parent-reported anxiety scores post-scan compared with baseline and pre-scan scores.

A repeated measures ANOVA with a Greenhouse–Geisser correction determined that there was a significant interaction effect of age group on parent-reported anxiety between baseline scores and pre-scan scores (F(1,79) = 14.09; *p* < 0.05), but not between pre-scan scores and post-scan scores (F(2,78) = 1.65; *p* > 0.05). The decrease in parent-reported anxiety between baseline and pre-scan was significantly higher in the younger group than in the older group, but this difference between groups was resolved in the comparison of pre-scan and post-scan reports. 

## 4. Discussion

The present research project aimed to evaluate the effectiveness of a novel smartphone application in preparing children for their upcoming MRI scans at home across different hospital settings. Developed in collaboration with clinical, technical, and commercial partners, the COSMO@home app incorporates a child-friendly design and learning goals based on psychological principles.

The findings of this study demonstrate the value of the COSMO@home app in preparing children for their MRI scans, with a high success rate of obtaining high-quality MRI images (95%) across both age groups (4–6 years and 7–10 years) and all participating hospitals, regardless of their specific contexts and workflows. Both children and parents expressed high satisfaction with the app, highlighting its positive reception.

Moreover, the results indicate that the app, when combined with minimal guidance that was child-friendly on the day of the scan, has the potential to be as effective as face-to-face training. The majority of participants (90%) required minimal additional preparation time (less than 5 min) on the day of the scan, in contrast with the average 30 to 60 min typically needed for face-to-face training [4,25,26]. These outcomes were consistent across both young children (4–6 years old) and older children (7–10 years old), indicating the app’s effectiveness across a broad age range. The brief preparation sessions mainly involved a small introduction, reassurance, and recapitulation of learning goals. The use of story elements from the game facilitated quick connection and knowledge reinforcement by the researchers. It is noteworthy that despite the shorter preparation time, the radiology departments experienced no disruptions in their regular workflows.

The involvement of parents is imperative for creating a consistent and confidence-building environment for children [27]. By including a parent module in the app, parents also received information about the MRI procedure and were better equipped to prepare their child for the appointment. The high level of parental satisfaction indicates that parents appreciated this method and found it valuable in addressing their own insecurity and helping them guide their children.

Parent-rated anxiety was utilized as a measure to assess a child’s fear regarding the upcoming MRI investigation. Therefore, it is essential to exercise caution in interpreting the observed decrease in anxiety, as it may not solely reflect a true decrease in the child’s anxious state but could also be influenced by the emotional state of the parent. That being said, the findings indicate that playing with the app led to a significant reduction in parent-rated anxiety, particularly in the youngest group of children (4–6 years old), whereas no significant reduction was observed in the older group (7–10 years old). The anxiety reduction in the younger group aligns with previous research highlighting their increased susceptibility to anxiety and the potential benefits of preparatory interventions in this age group [28]. The ability of the COSMO@home app to address the specific needs and anxieties of younger children could be attributed to its child-friendly design, engaging interface, and age-appropriate content. The interactive nature of the app likely provided a sense of familiarity and comfort to the younger children, facilitating their ability to cope with the upcoming MRI scan.

On the other hand, the lack of a significant reduction in anxiety among older children might be attributed to several factors. It is possible that older children already possess a certain level of understanding and coping mechanisms [17], rendering the app’s content and features less impactful in reducing their anxiety levels. Additionally, older children might have different sources of anxiety or concerns related to the MRI scan that were not effectively addressed by the app alone [18]. This highlights the importance of considering age-specific factors and tailoring interventions to meet the unique needs of different age groups.

Further research is warranted to investigate the reasons behind the differential effects of the app on anxiety reduction among different age groups. This could involve exploring the specific anxiety triggers and concerns of older children and adapting the app’s content to better address these factors. Additionally, it would be valuable to assess the potential influence of individual characteristics, such as temperament and previous experiences, on the effectiveness of the app in reducing anxiety across age groups.

Despite the lack of significant anxiety reduction in the older group, it is important to note that the overall satisfaction levels among both children and parents remained high. This suggests that the app, although not leading to a significant reduction in anxiety for older children, still provided value in terms of information provision, familiarization with the procedure, and facilitating communication between parents and children regarding the upcoming MRI scan.

The finding that the app effectively reduced anxiety in the youngest age group is of great clinical importance, as these children are at a higher risk of being sedated during an MRI scan [29]. Limited data suggest that a significant percentage of pediatric MRI scans involve sedation or general anesthesia (GA), with rates ranging from 70% to 85% for children below 6 years and 25% to 75% for children between 7 and 12 years [16,30]. If we apply these rates to our study group, it can be estimated that 23 to 28 children in the 4–6 years age group would have required sedation or GA, compared with the 4 children in our study whose scans were scheduled for another sedated MRI scan. Hence, the combination of the COSMO@home app with limited face-to-face preparation resulted in a reduction in sedation and GA in pediatric scans by 83–86% in children between 4 and 6 years. Extrapolating this reasoning to our older group, it is estimated that 12 to 37 children between 7 and 10 years would have required sedation. In our study, all children in this age group successfully underwent an awake scan when prepared with the COSMO@home app and limited face-to-face guidance. Nevertheless, it is imperative to recognize that our inclusion criteria might have introduced selection bias by excluding children with severe developmental disorders. Such children are representative of the broader clinical population. Future studies should explore the potential benefits of our approach for these children. Nevertheless, the results suggest that sedation or GA can be avoided for the majority of MRI scans in children as young as four years old, which is in line with findings of studies using other preparation methods [28,31,32].

While the results are promising, it is important to acknowledge that not all children were successfully prepared with the app, necessitating further research to identify predictive characteristics of non-responders. Additionally, future studies should include control groups to confirm the effectiveness of the app in reducing anxiety in children and explore potential financial and organizational benefits for patients and hospitals.

When interpreting the results of this study, it is important to consider several limitations that may impact the generalizability and interpretation of the findings. First, the absence of a control group restricts our ability to draw definitive conclusions about the efficacy of the app and its potential cost and time savings compared with traditional MRI approaches in children. Despite our efforts to restrict communication between the study team and participants, the absence of a control group prevents us from excluding the possibility of latent support influencing the outcomes, potentially introducing a placebo effect in certain children. Establishing a matched control group in the context of pediatric MRI, which exhibits considerable variability, poses challenges. Future research that incorporates a control group can provide further insights into these aspects. Nonetheless, the current study contributes valuable information by demonstrating the feasibility of non-sedated MRI in young children with minimal human support.

Another limitation to consider is the potential influence of participating in a research project, which involves close contact with researchers, consistent reassurance, and accessible support channels. These factors may have influenced the perceptions of both children and parents regarding the upcoming MRI appointment. The methodology employed in this study does not allow for the isolation and assessment of possible placebo effects, which could have influenced the reported outcomes. Future research could employ more rigorous experimental designs, such as randomized controlled trials, to better understand the specific effects of the app beyond potential placebo effects.

Furthermore, it is important to acknowledge that the selection criteria for this study excluded children with severe developmental disorders or other medical contraindications. As a result, the findings may not be applicable to this particular group of children, who often present unique challenges in clinical care. Future research should aim to investigate the potential benefits and suitability of this approach for children with severe developmental disorders or medical contraindications, as they represent a population of significant clinical concern.

## 5. Conclusions

In summary, this study contributes to the growing body of evidence supporting the feasibility of awake MRI scans for young children when adequate preparation is provided. Furthermore, the utilization of innovative technologies, such as the COSMO@home app, shows potential in reducing the workload and time requirements for hospital staff. Further research is warranted to assess the clinical effectiveness of the app for a more diverse pediatric population and to explore its broader implications in pediatric radiology.

## Figures and Tables

**Table 1:** Minigames and learning goals.

Minigame	Learning Goal
Balance game	Practice lying still
Size comparison game	Become familiar with size of MRI scanner
Real size game	Become familiar with size of MRI scanner
Defend the magnet game	Understand metal is not allowed in the MRI room
Quiz game	Learn about MRI procedure
What is MRI movie	Learn about MRI procedure
Lying still game	Practice lying still
Sound memory game	Become familiar with MRI sounds
Scanning game	Practice timings

**Table 2:** Criteria for extra face-to-face preparation.

Item	Criteria
Scores in quiz game	Lower than 4 out of 8 right answers
Child-reported anxiety	Score higher than 2 out of 5
Researcher-reported anxiety	Score higher than 4 out of 10
Parent-reported willingness	Score lower than 6 out of 10
Trainer-reported willingness	Score lower than 6 out of 10

Note: extra face-to-face preparation was given by the researcher if one or more of the above criteria was met.

**Table 3:** Descriptive results and general findings.

Variable	All Ages N = 82	4–6 Years N = 33	7–10 Years N = 49
Age ^a^, yrs	7.3 ± 1.7	5.6 ± 0.6	8.4 ± 1.2
Age range, yrs	4–10	4–6	7–10
Previous MRI history, N°	33 (40%)	16 (49%)	17 (35%)
IV infusion, N°	27 (33%)	13 (39%)	17 (35%)
Play time ^a^	133.4 ± 72.7	156.5 ± 58.3	118.7 ± 77.4
App sessions ^a^	5.0 ± 3.4	4.8 ± 2.6	5.2 ± 3.9
Space missions ^a^	4.9 ± 2.4	5.2 ± 2.2	4.6 ± 2.5
Likeability app ^b^	4.3 ± 1.1	4.7 ± 0.7	4.0 ± 1.2
Ease level to play ^b^	3.8 ± 1.2	3.7 ± 1.3	3.8 ± 1.2
Additional preparations, N°			
None	43 (52%)	17 (55%)	25 (51%)
0–5 min	29 (35%)	11 (34%)	37%
5–10 min	7 (9%)	2 (6%)	5 (10%)
>10 min	1 (1%)	0 (0%)	1 (0%)
Positive emotional state at placement in MRI, N°	66 (80%)	26 (80%)	40 (82%)
Scan time, N°			
<10 min	2 (2%)	0 (0%)	2 (4%)
10–20 min	44 (54%)	20 (61%)	24 (49%)
20–30 min	19 (23%)	6 (18%)	13 (27%)
>30 min	13 (16%)	3 (9%)	10 (20%)
Successful scans, N°	78 (95%)	29 (88%)	49 (100%)
Aborted scans, N°	4 (5%)	4 (12%)	0 (0%)
Qualitative scans, N°	77 (94%)	29 (88%)	48 (98%)
Scan time as planned, N°	64 (78%)	25 (77%)	39 (80%)
Interrupted scans, N°	8 (10%)	3 (9%)	5 (10%)
Major movements of child, N°	24 (29%)	12 (36%)	12 (24%)
Positive emotional state after scan, N°	73 (92%)	27 (82%)	46 (94%)
Satisfaction with app as preparation ^c^	9.1 ± 1.1	9.1 ± 0.9	9.0 ± 1.2
App use next time ^c^	8.0 ± 2.8	8.4 ± 2.6	7.7 ± 2.8
Success of MRI procedure ^d^	9.0 ± 1.6	8.7 ± 1.9	9.2 ± 1.3
App successful as preparation ^d^	8.6 ± 1.8	8.8 ± 1.7	8.5 ± 1.9

^a^ Results displayed as means with standard deviations. ^b^ Answers by children on a scale from 0 to 5, with 5 as best possible outcome. ^c^ Answers by parents on a scale from 0 to 10, with 10 as best possible outcome. ^d^ Answers by researchers on a scale from 0 to 10, with 10 as best possible outcome.

**Table 4:** Mean anxiety scores of different respondents at different time points for the two age groups.

	Baseline		Pre-Scan		Post-Scan	
	4–6 Yrs	7–10 Yrs	4–6 Yrs	7–10 Yrs	4–6 Yrs	7–10 Yrs
Parents	5.8 ± 2.3	4.8 ± 2.8	3.9 * ± 2.6	4.3 ± 2.7	2.4 *^+^ ± 2.7	2.5 *^+^ ± 2.1
Researchers	/	/	2.0 ± 1.2	1.9 ± 1.7	1.0 ^+^ ± 1.6	0.9 ^+^ ± 1.1

Note: Parents’ and researchers’ scores are on a scale ranging from 0 to 10. * *p* < 0.001 compared with baseline score. ^+^ *p* < 0.001 compared with pre-scan score.

## Data Availability

Data are contained within the article.
